# 25-hydroxyvitamin D concentration is inversely associated with serum MMP-9 in a cross-sectional study of African American ESRD patients

**DOI:** 10.1186/1471-2369-12-24

**Published:** 2011-05-22

**Authors:** Haimanot Wasse, Francesca Cardarelli, Christine De Staercke, Craig Hooper, Emir Veledar, Idris Guessous

**Affiliations:** 1 Emory University, Division of Nephrology, Atlanta, GA, USA; 2 Emory University, Rollins School of Public Health, Department of Epidemiology, Atlanta, GA, USA; 3 Emory University, Division of Cardiology, Atlanta, GA, USA; 4 Centers for Disease Control and Prevention, Division of Blood Disorders, Atlanta, GA, USA; 5 Unit of Population Epidemiology, Division of primary care medicine, Department of community medicine, primary care and emergency medicine. Geneva University Hospital, Geneva, Switzerland; 6 Community Prevention Unit, University Institute of Social and Preventive Medicine (IUMSP), Lausanne University Hospital, Lausanne, Switzerland

## Abstract

**Background:**

Circulating 25-hydroxyvitamin D [25(OH)D] concentration is inversely associated with peripheral arterial disease and hypertension. Vascular remodeling may play a role in this association, however, data relating vitamin D level to specific remodeling biomarkers among ESRD patients is sparse. We tested whether 25(OH)D concentration is associated with markers of vascular remodeling and inflammation in African American ESRD patients.

**Methods:**

We conducted a cross-sectional study among ESRD patients receiving maintenance hemodialysis within Emory University-affiliated outpatient hemodialysis units. Demographic, clinical and dialysis treatment data were collected via direct patient interview and review of patients records at the time of enrollment, and each patient gave blood samples. Associations between 25(OH)D and biomarker concentrations were estimated in univariate analyses using Pearson's correlation coefficients and in multivariate analyses using linear regression models. 25(OH) D concentration was entered in multivariate linear regression models as a continuous variable and binary variable (<15 ng/ml and ≥15 ng/ml). Adjusted estimate concentrations of biomarkers were compared between 25(OH) D groups using analysis of variance (ANOVA). Finally, results were stratified by vascular access type.

**Results:**

Among 91 patients, mean (standard deviation) 25(OH)D concentration was 18.8 (9.6) ng/ml, and was low (<15 ng/ml) in 43% of patients. In univariate analyses, low 25(OH) D was associated with lower serum calcium, higher serum phosphorus, and higher LDL concentrations. 25(OH) D concentration was inversely correlated with MMP-9 concentration (r = -0.29, p = 0.004). In multivariate analyses, MMP-9 concentration remained negatively associated with 25(OH) D concentration (P = 0.03) and anti-inflammatory IL-10 concentration positively correlated with 25(OH) D concentration (P = 0.04).

**Conclusions:**

Plasma MMP-9 and circulating 25(OH) D concentrations are significantly and inversely associated among ESRD patients. This finding may suggest a potential mechanism by which low circulating 25(OH) D functions as a cardiovascular risk factor.

## Background

The chronic pro-inflammatory state of end-stage renal disease (ESRD) is associated with increased risk for cardiovascular disease and death [1-3]. Observational studies suggest an inverse association between circulating 25-hydroxyvitamin D [25(OH) D] concentration and serum inflammatory biomarkers. Low 25(OH)D concentrations are associated with elevated tumor necrosis factor-alpha (TNF-α) concentrations [4], and randomized controlled studies among critically ill patients and those with congestive heart failure report that vitamin D supplementation leads to significant reductions in interleukin 6 ( IL-6) and C-reactive protein (CRP) concentrations [5], and resulted in lower tumor necrosis factor-alpha (TNF-α) and increased anti-inflammatory IL-10 concentrations [6]. However, few studies have addressed the association between 25(OH)D concentration and increased inflammation on biomarkers of vascular remodeling. Matrix metalloproteinases (MMP's) are important in extracellular matrix remodeling, are major effector molecules of inflammatory cells, and play a key role in many vascular diseases, including hypertension and aneurysm formation [7,8]. Recent *in vitro *studies indicate that vitamin D down-regulates MMP-9 production by TNF-α [9] and suppresses production of MMP-7 and MMP-9 [10-12]. Moreover, MMP-2 and MMP-9 activation are associated with collagen deposition and cardiac fibrosis [13].

Previous studies document the high prevalence of 25(OH) D deficiency within the U.S. ESRD and general population [14,15], and the association of 25(OH)D deficiency with vascular disorders [16,17]. Therefore, examination of the relationship between circulating 25(OH)D and biomarkers of vascular remodeling is warranted, as identification of a link between 25(OH) D concentration and vessel remodeling may suggest a mechanism for vitamin D deficiency as a risk factor for cardiovascular disease, and with further study, may offer promising therapy to improve vascular function among chronic kidney disease and ESRD patients.

We sought to determine whether circulating 25(OH) D concentration is associated with serum markers of vascular remodeling and inflammation in a cross-sectional study of prevalent hemodialysis African American patients.

## Methods

### Study Population

Adult ESRD patients receiving in-center maintenance hemodialysis at one of six Emory University-affiliated Davita dialysis centers between September 2006-November 2008, were eligible to enroll in the study. The majority of patients within the Emory dialysis centers are African American; of 94 patients enrolled, 91 were AA, thus we limited our analyses to AA patients. Patients were excluded from the study for the following reasons: 1) presence of known malignancy or active vasculitis; 2) evidence of current infection or inflammation; or 3) current or recent use of steroids, calcineurin inhibitors or antimetabolite medications (methotrexate, azathioprine, 5-fluorouracil, mercaptopurine, sulfadiazine). These exclusion criteria were chosen because they provide inflammatory or anti-inflammatory therapy that could alter the association between vitamin D and inflammatory markers. Demographic, clinical and dialysis treatment data were collected via direct patient interview and review of patients records at the time of enrollment, and each patient gave baseline blood samples. The Institutional Review Board (IRB) of Emory University Medical Center approved the study protocol and informed consent was obtained from each patient prior to study enrollment. Human subjects procedures followed were in accordance with the ethical standards of the institutional IRB and with the Helsinki Declaration of 1975.

### Definition of variables

All assessments were conducted at a single baseline visit. We analyzed several covariates including age, gender, self-reported race, length of time on dialysis, body mass index (BMI), smoking status (never, former current), vascular access type (arteriovenous fistula, arteriovenous graft, central venous catheter), season of blood collection, and activated vitamin D treatment. Comorbidities included diabetes (defined by use of diabetes medications), hypertension, stroke or transient ischemic event, congestive heart failure, peripheral arterial disease (PAD), and HIV or AIDS. Results of calcium, phosphorus, intact PTH, albumin, hemoglobin, KT/V (used to quantify hemodialysis adequacy), total cholesterol, LDL and HDL cholesterol, and triglycerides obtained within two weeks of the baseline blood sample measurement were abstracted from the outpatient dialysis records and included in the analysis.

### Study Measurements

#### Serum Cytokine Measurement

All patients received hemodialysis treatment three times per week. Blood for the analysis of serum biomarkers was drawn specifically for this study protocol, and was obtained before a routine, midweek hemodialysis session within 5 days of study enrollment. After collection, serum and plasma were aliquoted and stored at -80 C for subsequent analysis.

All assays were performed on plasma as directed by the manufacturer and samples were centrifuged for 10 minutes at 10000rpm before testing. All the assays had quality controls, plasma controls and a non-plasma sample that was added to each plate. The dilution of the samples (always done with the Calibration diluent in which the Standard Cocktail was reconstituted ) depended on the individual assays and of the expected concentration range for the samples tested.

The Fluorokine^® ^MultiAnalyte Profiling (MAP) Kits from R&D Systems (Minneapolis, USA) were used to determine the concentration of analytes. The Fluorokine^® ^MAP MultiAnalyte Profiling Human MMP Base Kit (R&D Systems) was used to determine the matrix metalloproteinase 9 (MMP-9) and MMP-2 concentrations (the inter-assay variability is 3.0% and 6.2% respectively). Il-6 and Il-10 concentrations were measured with the Fluorokine^® ^MAP MultiAnalyte Profiling Human Base Kit A (R&D Systems) (the inter-assay variability is 4.6% and 5.2% respectively). The CardioPhase hsCRP reagents from Siemens Diagnostics (Dade Behring product) were used to determine the concentration of hsCRP in plasma samples on a BNII (nephelometry systems) System. The Internal Quality Controls (Controls SL/1 and SL/2 - lower-higher range of CRP) and Standard SL curve were run with the assay.

#### 25(OH)D Measurement

Serum 25(OH) D concentrations were measured using chemiluminescence immunoassay (Diasorin Inc, Stillwater, MD, USA; coefficient of variation over multiple runs 6.3-12.9%) by ARUP Laboratories (Salt Lake City, UT). 25(OH) D concentration was categorized using a cutoff of 15 ng/ml, the point below which there is an increased risk for cardiovascular events [18,19].

### Statistical Analysis

Categorical variables were reported by using the number or percent of observations. Continuous variables were expressed as means with standard deviations. For comparative evaluations, Chi-square tests (Fisher's exact test when appropriate) and student unpaired independent t tests were performed for categorical and continuous variables, respectively. Distribution figures and Kolmogorov-Smirnov tests were used to assess inflammatory marker normality. Non-normally distributed markers were successfully normalized by natural logarithmic transformation. We used figures plots to verify that relationships, if any, between biomarkers and 25(OH)D concentrations were linear. Associations between 25(OH)D and biomarker concentrations were first estimated in univariate analyses using Pearson's correlation coefficients and corresponding P values.

To adjust for possible confounding of these associations we then used multivariate linear regression models. Included in the models were previously reported confounders as well as variables significant at the 5% level from the univariate analyses. 25(OH) D concentration was first entered in multivariate linear regression models as a continuous variable. Adjusted associations between continuous vitamin D concentration and biomarkers were reported as beta regression coefficients where the null value (β = 0) represented no association. We then categorized 25(OH) D using a binary variable (<15 ng/ml and ≥15 ng/ml). Adjusted estimate concentrations of biomarkers were compared between 25(OH) D groups using analysis of variance (ANOVA) with SAS generalized linear model procedures. Finally, results were stratified by vascular access type. Two-tailed P values P < 0.05 were considered statistically significant. All analyses were performed using SAS version 9.2 (SAS Institute, Cary, NC, USA).

## Results

### Patient Characteristics

The characteristics of the entire study cohort and of those classified by serum 25(OH) D concentrations are summarized in Table 1. Lower 25(OH) D concentration was significantly associated with lower serum calcium, higher serum phosphorus, and higher LDL concentration. There were no significant differences in other characteristics between vitamin D status groups. Among the study cohort, all subjects were treated with activated (D3) vitamin D, with the exception of 17 subjects, who took no form of vitamin D (activated or nutritional), and 3 subjects who took only nutritional (D2) vitamin D. The characteristics of the cohort by vascular access type are summarized in Table 2. Vascular access type was significantly associated with diabetes, stroke or TIA, HIV or AIDS, 25(OH)D and hemoglobin concentrations.

**Table 1:** Characteristics of African American participants by circulating 25(OH) D concentration

Characteristic	All patients	25(OH) D < 15 ng/ml	25(OH) D ≥ 15 ng/ml	
	N = 91	N = 39	N = 52	p value
Age (years)	59.3 ± 12.4	56.4 ± 12.6	61.4 ± 12.0	0.05
Sex				0.27
Female	43 (47.3)	21 (48.5)	22 (51.2)	
Male	48 (52.7)	18 (37.5)	30 (62.5)	
Dialysis vintage (yrs)	5.7 ± 4.1	6.2 ± 4.5	5.3 ± 3.9	0.33
KT/V*	1.4 ± 0.3	1.4 ± 0.2	1.5 ± 0.4	0.38
Body mass index (kg/m2)				0.10
Under (<18.5)	1 (1.2)	0 (0.0)	1 (100.0)	
Normal (18.5 to <25)	23 (26.7)	6 (26.09)	17 (73.9)	
Overweight (25 to <30)	37 (43.0)	18 (48.6)	19 (51.4)	
Obese (> = 30)	25 (29.1)	14 (56.0)	11 (44.0)	
Smoking status				0.08
Never	55 (60.4)	28 (50.9)	27 (49.09)	
Former	23 (25.7)	7 (30.4)	16 (69.57)	
Current	13 (14.3)	4 (30.8)	9 (69.23)	
Vascular access type				0.34
AVF	38 (41.8)	13 (34.2)	25 (65.8)	
Graft	44 (48.4)	21 (47.7)	23 (52.3)	
Catheter	9 (9.0)	5 (55.6)	4 (44.4)	
Activated vitamin D treatment	39 (42.9)	27 (29.7)	44 (48.3)	0.08
				
**Comorbidities**				
Diabetes	45 (49.4)	23 (51.1)	22 (48.9)	0.11
Hypertension	89 (97.8)	38 (42.7)	51 (57.3)	0.84
Stroke or TIA	16 (17.6)	10 (62.5)	6 (37.5)	0.08
Congestive heart failure	21 (23.1)	9 (42.9)	12 (57.1)	0.99
Peripheral arterial disease	13 (14.3)	7 (53.9)	6 (46.1)	0.79
HIV or AIDS	3 (3.3)	0 (0.0)	3 (100.0)	0.26
				
**Laboratory tests**				
25(OH)D level (ng/ml)	18.8 ± 9.6	11.1 ± 1.9	24.5 ± 9.0	
Calcium (mg/dl)	8.8 ± 0.8	8.5 ± 0.9	9.0 ± 0.8	<0.001
Phosphorus (mg/dl)	5.3 ± 2.0	5.8 ± 1.8	5.0 ± 2.1	0.05
intact PTH (pg/dl)	478.5 ± 537.09	569.9 ± 693.5	409.9 ± 373.6	0.16
Albumin (g/dl)	3.9 ± 0.4	3.9 ± 0.1	3.9 ± 0.1	0.74
Alkaline Phosphatase (U/L)	143.0 ± 113.0	164.9 ± 53.0	126.5 ± 66.7	0.15
Hemoglobin (g/l)	12.1 ± 1.4	12.0 ± 1.5	12.2 ± 1.3	0.63
Total Cholesterol (mg/dl)	154.4 ± 32.8	160.6 ± 35.6	149.7 ± 29.9	0.13
Triglycerides (mg/dl)	133.2 ± 75.3	135.6 ± 66.0	131.3 ± 82.6	0.79
HDL cholesterol (mg/dl)	48.1 ± 19.4	44.4 ± 15.8	51.0 ± 21.6	0.12
LDL cholesterol (mg/dl)	79.1 ± 31.8	87.1 ± 36.5	72.8 ± 26.3	0.04

Results are expressed as number (%) or mean ± standard deviation

*KT/V = dialysis adequacy

**Table 2:** Characteristics of African American participants by vascular access type

Characteristic	Arteriovenous fistula (AVF)	Arteriovenous graft (AVG)	Catheter	
	N = 38	N = 44	N = 9	p value
Age (years)	57.2 ± 13.0	60.8 ± 12.4	60.7 ± 9.6	0.41
Sex				0.07
Female	13 (34.2)	26 (29.9)	4 (44.4)	
Male	25 (65.8)	18 (40.9)	5 (55.6)	
Dialysis vintage (yrs)	4.9 ± 3.4	6.4 ± 4.5	6.1 ± 5.2	0.25
KT/V*	1.5 ± 0.4	1.4 ± 0.3	1.4 ± 0.2	0.49
Body mass index (kg/m2)				0.67
Under (<18.5)	0 (0.0)	0 (0.0)	1 (11.1)	
Normal (18.5 to <25)	10 (28.6)	13 (30.9)	0 (0.0)	
Overweight (25 to <30)	13 (37.1)	18 (42.9)	6 (66.7)	
Obese (> = 30)	12 (34.3)	11 (26.2)	2 (22.2)	
Smoking status				0.45
Never	21 (55.3)	31 (70.5)	3 (33.3)	
Former	8 (21.0)	10 (22.7)	5 (55.6)	
Current	9 (23.7)	3 (6.8)	1 (11.1)	
Activated vitamin D treatment				
	29 (76.3)	34 (77.3)	8 (88.9)	0.70
**Comorbidities**				
Diabetes	13 (34.2)	26 (59.1)	6 (66.7)	0.02
Hypertension	37 (97.4)	44 (100.0)	8 (88.9)	0.48
Stroke or TIA	2 (5.3)	11 (25.0)	3 (33.3)	0.03
Congestive heart failure	10 (26.3)	10 (22.7)	1 (11.1)	0.62
Peripheral arterial disease	4 (10.5)	8 (18.2)	2 (22.2)	0.60
HIV or AIDS	0 (0.0)	1 (2.3)	2 (22.2)	<0.01
				
**Laboratory tests**				
25(OH)D level (ng/ml)	21.6 ± 11.3	16.7 ± 7.3	17.1 ± 9.7	0.05
Calcium (mg/dl)	8.9 ± 0.8	8.7 ± 0.9	8.8 ± 1.1	0.65
Phosphorus (mg/dl)	5.5 ± 2.2	5.1 ± 1.6	5.5 ± 2.7	0.63
intact PTH (pg/dl)	401.6 ± 268.2	515.1 ± 693.0	624.4 ± 668.8	0.44
Albumin (g/dl)	4.1 ± 0.4	6.1 ± 0.2	3.7 ± 0.2	0.63
Alkaline Phosphatase (U/L)	115.4 ± 57.0	171.2 ± 146.6	120.9 ± 67.1	0.07
Hemoglobin (g/l)	12.13 ± 1.1	12.1 ± 1.6	11.0 ± 1.6	0.04
Total Cholesterol (mg/dl)	152.1 ± 31.2	153.7 ± 31.3	168.9 ± 46.4	0.42
Triglycerides (mg/dl)	132.4 ± 68.5	136.3 ± 82.1	121.4 ± 77.5	0.88
HDL cholesterol (mg/dl)	47.4 ± 20.5	48.6 ± 17.1	48.9 ± 27.2	0.96
LDL cholesterol (mg/dl)	79.3 ± 28.7	75.4 ± 32.3	95.6 ± 41.4	0.27

Results are expressed as number (%) or mean ± standard deviation

*KT/V = dialysis adequacy

### Serum 25(OH) D Concentrations

Mean 25(OH) D concentration was 18.8 ± 9.6 ng/ml. Thirty-nine (42.9%) subjects had a 25(OH) D concentration <15 ng/ml (Table 1). The mean serum 25(OH) D concentration of subjects in the higher (≥15 ng/ml) and lower (<15 ng/ml) vitamin D category were 24.5 ± 9.0 ng/ml and 11.1 ± 1.9 ng/ml, respectively.

### Serum Inflammatory Markers

#### Continuous 25(OH) D

**Figure 1:**
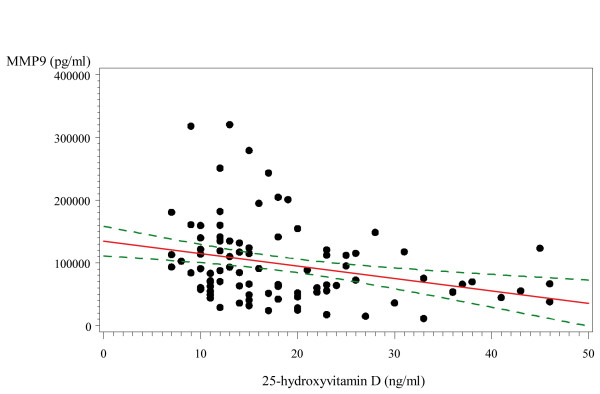
**Correlation between serum 25(OH)D and MMP-9 concentration**. Pearson correlation coefficient -0.29, P value = 0.004 Note: Continuous line represents regression line and dashed lines represent 95% confidence intervals

25(OH) D concentration was inversely and significantly correlated with MMP-9 concentration (r = -0.29, p = 0.004) (Figure 1), while the remaining biomarkers were not associated with 25(OH) D by correlation (Pearson) analysis.

#### Categorical 25(OH) D

The distribution of serum inflammatory markers by 25(OH) D concentration are shown in Table 3. In the univariate analyses and compared to patients with 25(OH)D ≥15 ng/ml, MMP-9 and CRP concentrations were significantly greater among subjects with 25(OH)D < 15 ng/ml. The remaining inflammatory markers were not associated with 25(OH)D categories.

**Table 3:** Distribution of inflammatory factors by serum 25(OH)D level

	All patients	25(OH) D <15 ng/ml	25(OH) D ≥ 15 ng/ml	P value
TNF- α (pg/ml)	8.7 ± 3.5	8.1 ± 2.9	9.0 ± 3.9	0.2
MMP-9 (pg/ml)	97517 ± 66188	115566 ± 66626	83981 ± 59392	0.01
MMP-2 (pg/ml)	548224 ± 156828	537353 ± 182886	488938 ± 165330	0.2
CRP (mg/L)	8.8 ± 16.0	10.5 ± 19.0	7.7 ± 13.0	0.03
IL-10 (pg/ml)	0.5 ± 0.6	0.4 ± 0.4	0.5 ± 0.6	0.6

Results are expressed as mean ± standard deviation

**Figure 2:**
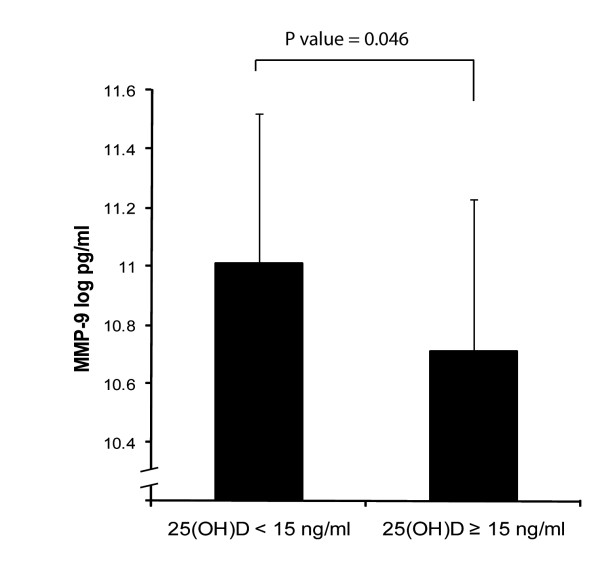
Difference in Log Concentration of MMP-9 by 25(OH)D group

We compared the difference in MMP-9 concentration between the lowest and highest 25(OH) D groups (Figure 2). Compared to the highest group and after adjustment for smoking, gender, BMI, age, diabetes, season and activated vitamin D treatment, the log MMP-9 concentration was greater in the lowest 25(OH)D group (P = 0.046). For every unit increase in 25(OH) D concentration, there was a log decrease in pro-inflammatory MMP-9 concentration of 0.018 pg/ml (p = 0.03), while there was a log increase in anti-inflammatory IL-10 concentration of 0.02 pg/ml (p = 0.04) (Table 4).

**Table 4:** Adjusted* multivariate associations between 25(OH) D concentration and inflammatory markers

	Model 1		Model 1 + vitamin D treatment adjustment		Model 1 + vitamin D treatment + season adjustment	
	
	**Beta coefficient **± **standard ** error	P value	**Beta coefficient **± **standard ** error	P value	**Beta coefficient **± **standard ** error	P value
Log TNF-α (pg/ml)	-0.003 ± 0.006	0.5	-0.002 ± 0.006	0.6	-0.003 ± 0.006	0.5
Log MMP-9 (pg/ml)	-0.017 ± 0.008	0.04	-0.019 ± 0.008	0.02	-0.018 ± 0.008	0.03
Log MMP-2 (pg/ml)	-0.003 ± 0.004	0.5	-0.004 ± 0.004	0.3	-0.004 ± 0.004	0.3
Log CRP (mg/ml)	-0.013 ± 0.015	0.3	-0.014 ± 0.014	0.4	-0.013 ± 0.014	0.3
Log IL-10 (pg/ml)	0.022 ± 0.010	0.04	0.021 ± 0.010	0.04	0.021 ± 0.010	0.04

Model 1 is adjusted for smoking, gender, BMI, age, and diabetes

Upon stratification of vascular access type (arteriovenous fistula (AVF) vs. arteriovenous graft (AVG)), it appears that the association between 25(OH) D concentration and MMP-9 was mostly driven by AVG's, as 25(OH)D and MMP-9 and IL-10 no longer remained associated among AVF users. In contr ast, for every unit increase in 25(OH) D concentration, the log concentration of MMP-9 decreased by 0.052 pg/ml (P = 0.002) among patients using an AVG, while the association of IL-10 to 25(OH) D no longer remained significant among AVG users (Table 5).

**Table 5:** Adjusted* associations between vitamin D level and inflammatory markers, stratified by vascular groups

	Arteriovenous fistula	P value	Arteriovenous graft	P value
Log MMP-9 (pg/ml)	0.004 ± 0.010	0.7	-0.052 ± 0.016	0.002

Log IL-10 (pg/ml)	0.016 ± 0.010	0.1	0.041 ± 0.023	0.08

*Adjusted for smoking, gender, BMI, age, diabetes, season and activated vitamin D treatment

Results are expressed as beta coefficient + standard error

## Discussion

Our results indicate that plasma MMP-9 expression is inversely and independently associated with circulating 25(OH) D concentration among ESRD patients. We also found anti-inflammatory IL-10 concentration to be associated with 25(OH) D concentration. This is the first report to our knowledge to assess the relationship of 25(OH)D concentration with MMP-9 and IL-10 in an ESRD population, and may help to shed light on mechanisms by which vitamin D deficiency is associated with an increased risk of atherosclerosis and cardiovascular disease.

Our results are consistent with previous data among non-ESRD populations that report a strong association between 25(OH) D deficiency and elevated markers of vascular remodeling: Vitamin D supplementation of vitamin D deficient, healthy British Bangladeshi adults resulted in significant reductions in plasma MMP-9 and serum CRP concentrations [20]. Vitamin D supplementation of congestive heart failure patients significantly increased serum concentrations of anti-inflammatory cytokine IL-10 and suppressed TNF-α synthesis over a nine month period [6]. Further, previous reports show that anti-inflammatory IL-10 is increased by vitamin D supplementation *in vitro *and in mice [11]. Vitamin D acts on dendritic cells, T-cells, and B-cells to enhance IL-10 expression [21-23].

While others have reported reductions in CRP concentrations following vitamin D supplementation [5,6,24], and negative correlations between serum 25(OH)D concentration and TNF-α concentrations [4], this was not observed in our study, possibly due to our sample size or that a greater differential in 25(OH)D concentration may be needed in order to observe an association with these biomarkers. Whilst we identified associations between both MMP-9 and IL-10 with 25(OH) D concentration, we also observed several important negative findings in this cross-sectional study following multivariate adjustment. First, while PTH concentrations were greater among patients with 25(OH)D < 15 ng/dL, they were not significantly different among the vitamin D cohorts, as we would have expected to observe, possibly due to our relatively small sample size. Second, there was no significant association observed between 25(OH) D and CRP, the most common biomarker of inflammation. This may be due to our relatively small sample size, however, other cross-sectional reports among non-ESRD populations have also failed to find an association between 25(OH)D concentration and CRP concentration following multivariate analysis, supporting our conclusion [4,14,18]. We did not observe elevations in MMP-9 and CRP concentrations as large as those reported previously in comparable patients [25], likely because MMP-9 gene expression is upregulated by several factors that were not included in our analysis. Finally, while MMP-2 concentrations tended to be higher with 25(OH)D concentrations <15 ng/dL, this did not reach significance, likely due to our sample size.

MMP's promote the remodeling of connective tissue and basement membranes via degradation of collagen, and act as important regulatory molecules in inflammation and vascular diseases. MMP-9 is released by neutrophils and is a key effector molecule of inflammatory cells, aiding migration of inflammatory leukocytes through tissue barriers, lysing protein substrates, modulating smooth muscle cell migration, and promoting angiogenesis [26,27]. MMP's also regulate inflammation by directly and indirectly acting on pro-inflammatory cytokines, such as TNF-α and TGF-β, to control chemokine activity [27]. 25(OH)D may act to reduce MMP-9 in several ways. An increase in IL-10 concentration, as we observed, may also act to suppress MMP-9 secretion [28]. Pulmonary tuberculosis *in vitro *studies suggest that vitamin D blunts MMP-9 expression by inhibition of c-Jun-N-terminal kinase(JNK) and NFkappaB signaling cascades [9]. MMP-9 concentrations are increased in the setting of several chronic inflammatory diseases, and MMP elevations are associated with abdominal aortic aneurysm rupture [29] and acute myocardial infarction [30]. These findings suggest that the risk for acquiring or further progression of these disorders may be positively impacted by limiting MMP concentrations.

Another important finding of our study is that vascular access type modifies the relationship between plasma MMP-9 and circulating 25(OH) D concentrations. When we stratified AVF versus AVG, the association between MMP-9 and 25(OH) D concentrations no longer remained significant among AVF patients, whereas the relationship remained significant among patients using an AVG, suggesting that MMP-9 concentrations in AVF patients were too low for a relationship with 25(OH)D to be detected. This may occur because the prosthetic material within an AVG may induce increased systemic inflammation. This finding is supported by data examining the role of vascular access type on systemic inflammation among prevalent HD patients, which noted that CRP concentrations were significantly lower among autogenous AVF users compared with patients using a prosthetic AVG [31].

Limitations of this study should be considered. First, our relatively small sample size may affect our results, however the 25(OH) D concentrations observed among our subjects are comparable to those reported among larger ESRD patient cohorts, and the observed inverse association between 25(OH) D and MMP-9 has been reported in a larger, non-renal patient cohort [18]. Second, the cross-sectional design of our study does not allow for causal inference. We cannot exclude the possibility that an association exists between 25(OH) D and the additional inflammatory biomarkers examined in our study, but due to our relatively small sample size, we may be underpowered to detect it. For example, the power to detect a statistical difference, if any, between 25(OH)D groups was less than 50% for TNF-α and MMP-2. Third, although we have attempted to reduce the effect of confounding by limiting our study population to include only patients free of infection or inflammation, residual confounding inherent to the cross-sectional design cannot be excluded.

## Conclusions

In conclusion, the results of our study reveal a new finding of a significant inverse relationship between plasma MMP-9 and circulating 25(OH) D concentrations among AA ESRD patients, and suggest that there may be value in measuring 25(OH) D concentrations in ESRD patients. In addition, we observed a positive association between 25(OH)D concentration and anti-inflammatory IL-10 concentration. Our findings extend data obtained from previous studies in linking low vitamin 25(OH)D concentrations to elevated concentrations of the cardiovascular risk marker, circulating MMP-9, in ESRD patients. Given the high prevalence of nutritional vitamin D deficiency and cardiovascular disease among ESRD patients, our findings suggest that future studies are warranted which further characterize the relationship between nutritional vitamin D therapy and vascular remodeling biomarker synthesis.

## Competing interests

The authors declare that they have no competing interests.

## Authors' contributions

HW: design of protocol, evaluation of data, draft of the manuscript. IG: statistical analyses, collaborating in drafting of the manuscript. FC: data collection, preparation of database. CDE: laboratory analyses, immunoassays. CH: laboratory analyses, immunoassays. EV: preparation of database, statistical analyses. All authors read and approved the final manuscript

## Pre-publication history

The pre-publication history for this paper can be accessed here:

http://www.biomedcentral.com/1471-2369/12/24/prepub

## References

[B1] Segarra A Chacon P Martinez-Eyarre C Argelaguer X Vila J Ruiz P Fort J Bartolome J Camps J Moliner E Circulating levels of plasminogen activator inhibitor type-1, tissue plasminogen activator, and thrombomodulin in hemodialysis patients: biochemical correlations and role as independent predictors of coronary artery stenosis J Am Soc Nephrol 2001 12 1255 1263 11373350 10.1681/ASN.V1261255

[B2] Yeun JY Levine RA Mantadilok V Kaysen GA C-Reactive protein predicts all-cause and cardiovascular mortality in hemodialysis patients Am J Kidney Dis 2000 35 469 476 10.1016/S0272-6386(00)70200-9 10692273

[B3] Zoccali C Benedetto FA Mallamaci F Tripepi G Fermo I Foca A Paroni R Malatino LS Inflammation is associated with carotid atherosclerosis in dialysis patients. Creed Investigators. Cardiovascular Risk Extended Evaluation in Dialysis Patients J Hypertens 2000 18 1207 1213 10.1097/00004872-200018090-00006 10994751

[B4] Peterson CA Heffernan ME Serum tumor necrosis factor-alpha concentrations are negatively correlated with serum 25(OH)D concentrations in healthy women J Inflamm (Lond) 2008 5 10 10.1186/1476-9255-5-10 PMC2503979 18652680

[B5] Van den Berghe G Van Roosbroeck D Vanhove P Wouters PJ De Pourcq L Bouillon R Bone turnover in prolonged critical illness: effect of vitamin D J Clin Endocrinol Metab 2003 88 4623 4632 10.1210/jc.2003-030358 14557432

[B6] Schleithoff SS Zittermann A Tenderich G Berthold HK Stehle P Koerfer R Vitamin D supplementation improves cytokine profiles in patients with congestive heart failure: a double-blind, randomized, placebo-controlled trial Am J Clin Nutr 2006 83 754 759 16600924 10.1093/ajcn/83.4.754

[B7] Aziz F Kuivaniemi H Role of matrix metalloproteinase inhibitors in preventing abdominal aortic aneurysm Ann Vasc Surg 2007 21 392 401 10.1016/j.avsg.2006.11.001 17484978 PMC2128752

[B8] Friese RS Rao F Khandrika S Thomas B Ziegler MG Schmid-Schonbein GW O'Connor DT Matrix metalloproteinases: discrete elevations in essential hypertension and hypertensive end-stage renal disease Clin Exp Hypertens 2009 31 521 533 10.3109/10641960802668730 19886850 PMC2875383

[B9] Bahar-Shany K Ravid A Koren R Upregulation of MMP-9 production by TNFalpha in keratinocytes and its attenuation by vitamin D J Cell Physiol 2010 222 729 737 20020446 10.1002/jcp.22004

[B10] Anand SP Selvaraj P Effect of 1, 25 dihydroxyvitamin D(3) on matrix metalloproteinases MMP-7, MMP-9 and the inhibitor TIMP-1 in pulmonary tuberculosis Clin Immunol 2009 133 126 131 10.1016/j.clim.2009.06.009 19615945

[B11] Coussens A Timms PM Boucher BJ Venton TR Ashcroft AT Skolimowska KH Newton SM Wilkinson KA Davidson RN Griffiths CJ 1alpha,25-dihydroxyvitamin D3 inhibits matrix metalloproteinases induced by Mycobacterium tuberculosis infection Immunology 2009 127 539 548 10.1111/j.1365-2567.2008.03024.x 19178594 PMC2729531

[B12] Tetlow LC Woolley DE Expression of vitamin D receptors and matrix metalloproteinases in osteoarthritic cartilage and human articular chondrocytes in vitro Osteoarthritis Cartilage 2001 9 423 431 10.1053/joca.2000.0408 11467890

[B13] Rahman A Hershey S Ahmed S Nibbelink K Simpson RU Heart extracellular matrix gene expression profile in the vitamin D receptor knockout mice J Steroid Biochem Mol Biol 2007 103 416 419 10.1016/j.jsbmb.2006.12.081 17275288

[B14] Melamed ML Michos ED Post W Astor B 25-hydroxyvitamin D levels and the risk of mortality in the general population Arch Intern Med 2008 168 1629 1637 10.1001/archinte.168.15.1629 18695076 PMC2677029

[B15] Wolf M Shah A Gutierrez O Ankers E Monroy M Tamez H Steele D Chang Y Camargo CA Jr Tonelli M Thadhani R Vitamin D levels and early mortality among incident hemodialysis patients Kidney Int 2007 72 1004 1013 10.1038/sj.ki.5002451 17687259

[B16] Forman JP Giovannucci E Holmes MD Bischoff-Ferrari HA Tworoger SS Willett WC Curhan GC Plasma 25-hydroxyvitamin D levels and risk of incident hypertension Hypertension 2007 49 1063 1069 10.1161/HYPERTENSIONAHA.107.087288 17372031

[B17] Giovannucci E Liu Y Hollis BW Rimm EB 25-hydroxyvitamin D and risk of myocardial infarction in men: a prospective study Arch Intern Med 2008 168 1174 1180 10.1001/archinte.168.11.1174 18541825 PMC3719391

[B18] de Boer IH Kestenbaum B Shoben AB Michos ED Sarnak MJ Siscovick DS 25-Hydroxyvitamin D Levels Inversely Associate with Risk for Developing Coronary Artery Calcification J Am Soc Nephrol 2009 10.1681/ASN.2008111157 PMC2723983 19443637

[B19] Wang Y Krishnamoorthy M Banerjee R Zhang J Rudich S Holland C Arend L Roy-Chaudhury P Venous stenosis in a pig arteriovenous fistula model--anatomy, mechanisms and cellular phenotypes Nephrol Dial Transplant 2008 23 525 533 18037619 10.1093/ndt/gfm547

[B20] Timms PM Mannan N Hitman GA Noonan K Mills PG Syndercombe-Court D Aganna E Price CP Boucher BJ Circulating MMP9, vitamin D and variation in the TIMP-1 response with VDR genotype: mechanisms for inflammatory damage in chronic disorders? QJM 2002 95 787 796 10.1093/qjmed/95.12.787 12454321

[B21] Barrat FJ Cua DJ Boonstra A Richards DF Crain C Savelkoul HF de Waal-Malefyt R Coffman RL Hawrylowicz CM O'Garra A In vitro generation of interleukin 10-producing regulatory CD4(+) T cells is induced by immunosuppressive drugs and inhibited by T helper type 1 (Th1)- and Th2-inducing cytokines J Exp Med 2002 195 603 616 10.1084/jem.20011629 11877483 PMC2193760

[B22] Adorini L Intervention in autoimmunity: the potential of vitamin D receptor agonists Cell Immunol 2005 233 115 124 10.1016/j.cellimm.2005.04.013 15936743

[B23] Heine G Niesner U Chang HD Steinmeyer A Zugel U Zuberbier T Radbruch A Worm M 1,25-dihydroxyvitamin D(3) promotes IL-10 production in human B cells Eur J Immunol 2008 38 2210 2218 10.1002/eji.200838216 18651709

[B24] Alborzi P Patel NA Peterson C Bills JE Bekele DM Bunaye Z Light RP Agarwal R Paricalcitol reduces albuminuria and inflammation in chronic kidney disease: a randomized double-blind pilot trial Hypertension 2008 52 249 255 10.1161/HYPERTENSIONAHA.108.113159 18606901

[B25] Preston GA Barrett CV Alcorta DA Hogan SL Dinwiddie L Jennette JC Falk RJ Serum matrix metalloproteinases MMP-2 and MMP-3 levels in dialysis patients vary independently of CRP and IL-6 levels Nephron 2002 92 817 823 10.1159/000065464 12399626

[B26] Jorde R Haug E Figenschau Y Hansen JB Serum levels of vitamin D and haemostatic factors in healthy subjects: the Tromso study Acta Haematol 2007 117 91 97 10.1159/000097383 17135721

[B27] Parks WC Wilson CL Lopez-Boado YS Matrix metalloproteinases as modulators of inflammation and innate immunity Nat Rev Immunol 2004 4 617 629 10.1038/nri1418 15286728

[B28] Lacraz S Nicod LP Chicheportiche R Welgus HG Dayer JM IL-10 inhibits metalloproteinase and stimulates TIMP-1 production in human mononuclear phagocytes J Clin Invest 1995 96 2304 2310 10.1172/JCI118286 7593617 PMC185881

[B29] Wilson WR Anderton M Choke EC Dawson J Loftus IM Thompson MM Elevated plasma MMP1 and MMP9 are associated with abdominal aortic aneurysm rupture Eur J Vasc Endovasc Surg 2008 35 580 584 10.1016/j.ejvs.2007.12.004 18226564

[B30] Hlatky MA Ashley E Quertermous T Boothroyd DB Ridker P Southwick A Myers RM Iribarren C Fortmann SP Go AS Matrix metalloproteinase circulating levels, genetic polymorphisms, and susceptibility to acute myocardial infarction among patients with coronary artery disease Am Heart J 2007 154 1043 1051 10.1016/j.ahj.2007.06.042 18035073

[B31] Movilli E Brunori G Camerini C Vizzardi V Gaggia P Cassamali S Scolari F Parrinello G Cancarini GC The kind of vascular access influences the baseline inflammatory status and epoetin response in chronic hemodialysis patients Blood Purif 2006 24 387 393 10.1159/000093681 16755161

